# Polyphenol-Rich Beverages and Mental Health Outcomes

**DOI:** 10.3390/antiox12020272

**Published:** 2023-01-25

**Authors:** Agnieszka Micek, Joanna Jurek, Marcin Owczarek, Ida Guerrera, Sebastiano Alfio Torrisi, Sabrina Castellano, Giuseppe Grosso, Ali A. Alshatwi, Justyna Godos

**Affiliations:** 1 Institute of Nursing and Midwifery, Faculty of Health Sciences, Medical College, Jagiellonian University, 30-688 Krakow, Poland; 2 APC Microbiome Ireland, University College Cork, T12 K8AF Cork, Ireland; 3 School of Psychology, Ulster University, Coleraine BT52 1SA, UK; 4 Department of Biomedical and Biotechnological Sciences, University of Catania, 95123 Catania, Italy; 5 Department of Educational Sciences, University of Catania, 95124 Catania, Italy; 6 Center for Human Nutrition and Mediterranean Foods (NUTREA), University of Catania, 95123 Catania, Italy; 7 Department of Food Science and Nutrition, College of Food and Agricultural Sciences, King Saud University, Riyadh 11451, Saudi Arabia

**Keywords:** polyphenols, coffee, tea, wine, beer, citrus juice, mental health, sleep quality, depressive symptoms, perceived stress

## Abstract

Emerging evidence suggests that diets rich in plant-based foods and beverages may exert plausible effects on human health tackling the risk of chronic diseases. Although the data are promising for numerous outcomes, including cardiovascular diseases, the data on mental health are limited. The aim of this study was to investigate the association between individual polyphenol-rich beverages intake and mental health outcomes, such as perceived stress, depressive symptoms, and sleep quality, among adult individuals living in the Mediterranean area. The demographic and dietary characteristics of a sample of 1572 adults living in southern Italy were analysed. Multivariate logistic regression analyses, controlling for confounding factors, were used to calculate odds ratios (ORs) and 95% confidence intervals (CIs) of the association between individual polyphenol-rich and alcoholic beverages containing polyphenols and mental health outcomes. The multivariate model adjusted for background covariates and the Mediterranean diet showed that individuals with a moderate intake (up to 1 cup/glass per day) of coffee and tea were less likely to have high perceived stress (OR = 0.61, 95% CI: 0.45–0.84) and depressive symptoms (OR = 0.56, 95% CI: 0.39–0.80), respectively. Furthermore, regular coffee and moderate/regular red wine drinkers were less likely to have depressive symptoms (OR = 0.72, 95% CI: 0.54–0.95 and OR = 0.74, 95% CI: 0.54–0.99, respectively). No significant associations were retrieved for the intake of polyphenol-rich and alcoholic beverages and sleep quality. In conclusion, the results of the present study suggest that polyphenol-rich beverages may be associated with mental health, in terms of depressive symptoms and perceived stress. Nonetheless, further research exploring how the polyphenol-rich beverages impact brain health and what the optimal patterns of consumption are for different populations are warranted.

## 1. Introduction

Dietary habits and food choices are considered among the most important behavioral risk factors for non-communicable diseases worldwide [1]. The Global Burden of Disease Study (GBD) estimated that in 2017 dietary risk factors contributed to 11 million deaths globally [2]. Most of the existing summaries of scientific literature corroborate the evidence of some key drivers of diet quality, such as the high consumption of fiber-rich foods (including fruits, vegetables, whole grains, and legumes), the adequate intake of dairy products, the limited consumption of red/processed meat and sodium, and the avoidance of trans-fatty acids and refined added sugars [3]. Such features, easily applicable at a global level, would play a major role in reducing the burden of cardiovascular diseases, metabolic disorders, and certain cancers [2]. Emerging evidence suggests that diet might also exert far more complex effects in the human body, being potentially responsible for low-grade immune responses and all conditions potentially related to chronic inflammation [4]. Among the most intriguing hypotheses, dietary risks have been related to brain health [5]. While the influence of the brain toward the gastroenteric system is well-known, a growing number of studies suggests that dietary factors may be associated with various mental health outcomes, such as depressive symptoms, perceived stress, and sleep disorders [5,6]. These conditions have been estimated to be the leading causes of years lived with disability worldwide [7], while also being interconnected with each other and associated with several non-communicable diseases, including cardio-metabolic conditions and dementias [8]. Thus, it is of paramount interest to understand whether dietary factors may have played a role in the sudden increase in the burden of mental conditions and whether the adoption of specific dietary patterns could tackle the increasing trend.

Plant-based foods are rich in fiber, but a large body of scientific literature supports the hypothesis that other molecules, such as antioxidant phytochemicals, may exert positive effects on human health [9]. Several meta-analyses demonstrated consistent findings on the association between a higher intake of dietary polyphenols and a lower risk of cardiovascular disease [10] and overall mortality [11], hypertension [12], type-2 diabetes [13], and certain cancers [14,15]. These findings suggest a potential role of plant-based foods, such as fruits, vegetables, whole grains, and legumes, in preventing non-communicable diseases and justify their inclusion in dietary recommendations. Furthermore, the consumption of plant-based beverages, such as coffee, tea, fruit juices, beer, and red and white wine, have demonstrated a potential role for the prevention of certain non-communicable diseases [9].

Among less studied outcomes, there is emerging evidence that polyphenols may exert a beneficial role in neuroprotection [16]. There are several mechanisms that could explain the potential effects of polyphenols in the central nervous system, including protection from neuroinflammation [17], improvement in vascular and endothelial brain health [18], an increase in antioxidant defenses of specific brain areas [19], and ultimately modulation of the gut microbiota profile providing peripheral effects via the gut–brain axis [20]. Recently, some studies showed an association between the intake of specific polyphenols and sleep quality and depressive symptoms [21,22]. However, although the number of studies reporting on the association between polyphenol-rich beverages and mental health outcomes is increasing, current evidence is still unclear. The aim of this study is to investigate the association between individual polyphenol-rich beverages intake and mental health outcomes, such as perceived stress, depressive symptoms, and sleep quality, among adult individuals living in the Mediterranean area.

## 2. Materials and Methods

### 2.1. Study Population

The Mediterranean healthy Eating, Aging, and Lifestyle (MEAL) study is an observational study aimed to evaluate the association between Mediterranean dietary and lifestyle habits and chronic diseases. A detailed protocol of this study is published elsewhere [23]. In brief, a sample of men and women aged 18 or more years old was randomly selected by using the list of registered records of local general practitioners. Participants were enrolled between 2014 and 2015 from the main districts of Catania, a city of southern Italy. Data were stratified by 10-year age groups and sex. In order to provide a specific relative precision of 5% (Type I error, 0.05; Type II error, 0.10), considering an anticipated 70% participation rate, the theoretical sample size was estimated to 1500 individuals. Out of 2405 subjects invited to participate in the study, 2044 accepted to participate in the study (response rate of 85%). The aims of the study were explained in detail to all participants, and written informed consent was provided. All the procedures were performed in agreement with the Declaration of Helsinki (1989) of the World Medical Association. The study protocol has been revised and authorized by the concerning ethical committee.

### 2.2. Data Collection

Face-to-face personal interviews were conducted and an electronic data collection was implemented with the use of tablet computers. All participants were provided with a paper copy of the questionnaire to better visualize the response options, but the final answers were registered by the interviewer personally. The following background data were collected: sex, age at recruitment, highest degree of education, physical activity level, and smoking status. Educational status was categorized as (i) low (primary/secondary), (ii) medium (high school), and (iii) high (university). International Physical Activity Questionnaires (IPAQ) was used to estimate the physical activity level [24]. According to IPAQ, physical activity was categorized as (i) low, (ii) moderate, and (iii) high. Smoking status was classified as being a non, former-, or current smoker. Anthropometric assessments were undertaken according to the standardized techniques [25]. Body height was measured with precision of 0.5 cm in barefoot participants with a right-angle triangle resting on the scalp, who were standing back to the wall and focusing their eyes straight ahead. Body mass index (BMI) was categorized as normal weight (BMI < 25 kg/m^2^), overweight (BMI 25 to 29.9 kg/m^2^), and obese (BMI ≥ 30 kg/m^2^).

### 2.3. Dietary Assessment

The dietary intake was assessed using two versions of food frequency questionnaires (FFQs), a long and a short version [26,27], whose validity and reliability have been previously tested in the Sicilian population. The consumption of seasonal foods refers to the intake during the period in which the food was available and then adjusted by its proportional intake in one year. Employing the determination of the food intake, the energy content as well as the macro- and micronutrients intake was calculated through a comparison with food composition tables of Council for Research in Agriculture and Analysis of Agricultural Economy (CREA) [28]. Phenol-Explorer database was used to estimate polyphenol intake, as previously reported in detail [29]. Specifically, the individual food consumption (in mL or g) was obtained for each participant of the study by following the standard portion sizes converted to 24 h intake; next, the databases were searched to obtain average values for the energy content, macro-, micronutrients, and polyphenols contained in the foods (per 100 mL or g). Finally, the energy, nutrient, and polyphenol intake from each food was calculated by multiplying the content of each variable by the daily consumption of each food [30]. FFQs with lacking data or unreliable intakes (<1000 or >6000 kcal/d) were excluded from the analyses (n = 198) leaving a total of 1846 individuals. The variables of interest were intake of tea, coffee (espresso/stovetop), red and white wine, beer, and fruit juice (fresh citrus juice). For each polyphenol-rich beverage type, three type-specific categories of intake [(i) no consumption, (ii) up to one cup/glass per day, and (iii) more than one cup/glass per day] were considered. The categorization was based on roughly tertiles of distribution and universally accepted standard servings for beverage consumption.

A literature-based score was used to calculate adherence to the Mediterranean diet [31]. Adherence to the Mediterranean diet was used as a proxy of diet quality and assessed using a literature-based scoring system. Briefly, two points were given to the highest category of consumption of food groups typical of the Mediterranean pattern (such as vegetables, fruits, legumes, cereals, fish), one point for the middle category and 0 points for the lowest category of consumption. Conversely, two points were given for the lowest category of consumption of foods not characteristic to the Mediterranean diet (such as meat and dairy products), one point for the middle category, and 0 points for the highest category of consumption. A better adherence was guaranteed by a moderate alcohol intake and regular use of olive oil. The final score comprises nine food categories with a score ranging from 0 points (lowest adherence) to 18 points (highest adherence); thus, individuals were categorized in the following tertiles: (i) low, (ii) medium, and (iii) high adherence to the Mediterranean diet.

### 2.4. Mental Health Status Assessment

Participants’ sleep quality was evaluated using the Pittsburgh sleep quality index (PSQI) [32]. The questionnaire consists of 19 items classified on a four-point scale (0–3) and clustered into seven domains (sleep quality, sleep latency, sleep duration, habitual sleep efficiency, sleep disturbance, use of sleeping medications, and daytime dysfunction). The item scores in each component were summed and converted into domain scores ranging from 0 (better) to 3 (worse) based on guidelines. The total PSQI score was obtained by calculating the sum of the seven domain scores. The global PSQI score ranges from 0 to 21 points, with a higher score indicating worse sleep quality. A result of <5 on global PSQI score is indicative of adequate sleep quality.

The Perceived Stress Scale (PSS) was used to evaluate stress symptoms [33]. In brief, PSS is a 14-item questionnaire used to measure perceived stress, i.e., how individuals perceive situations as stressful. Each question has answer options ranging from zero to four (i.e., 0 = never, 1 = almost never, 2 = sometimes, 3 = often, and 4 = always). The global score is the sum of the scores of these 14 items and the global score ranges from 0 (minimum) to 56 (maximum). The sex-specific median value was considered as cut-off point to define high or low perceived stress.

In order to evaluate depressive symptoms, the 20-item Center for the Epidemiological Studies of Depression Short Form (CES-D) was used [34]. Briefly, CES-D is commonly used to screen for depressive symptoms in the general population. Each item of the scale rates the frequency of each mood or symptom ‘during the past week’ on a 4-point scale ranging from 0 (rarely or none of the time [less than 1 day]) to 3 (most or all of the time [5–7 days]). A score is assigned by totaling all items (after reversing the positive mood items); total scores can range from 0 to 30, with higher scores suggesting greater severity of symptoms, and a score ≥ 16 indicating as having depressive symptoms [35,36]. After exclusion of individuals with missing data about the mental and sleep health assessment, a total sample of 1572 was included in the final analysis.

### 2.5. Statistical Analysis

Categorical variables were characterized by absolute numbers and relative frequencies (%). Continuous features were reported as means and standard deviations. The distribution of background characteristics and polyphenol intake were compared between predefined groups of polyphenol-rich beverage consumption. Differences in percentages of categorical variables across three groups were verified with Chi-squared test. Comparison of normally and not normally distributed continuous variables between defined groups of subjects were conducted by applying ANOVA and Kruskall–Wallis test, respectively. Odds ratios (ORs) and 95% confidence intervals (CIs) for association between categories of intake of individual polyphenol-rich beverages and mental health outcomes (including sleep quality, perceived stress, and depressive symptoms) were calculated based on fitted multiple logistic regression models. To test possible effect of confounding factors we used three levels of adjustment with different sets of covariates, including energy intake and all polyphenol-rich beverage intake (model 1), all background characteristics (model 2), and adherence to the Mediterranean diet as a proxy of diet quality (model 3). This allowed us to verify robustness of the findings and check whether the retrieved associations were independent of the potential confounders, including overall quality of the diet. The significance level was set at 0.05 and all reported *p*-values were based on two-sided tests. Analyses were performed using the SPSS version 27 (SPSS Inc., Chicago, IL, USA).

## 3. Results

The background characteristics of the study sample are provided in Table 1. The mean age was 46.6 years old, less than half were men (42.0%). The majority of participants were never smokers with an average BMI of 25.6, reporting occasional alcohol drinking. Educational and physical activity levels were quite equally distributed in the study sample. Roughly one third of the participants reported having depressive symptoms, perceived stress, and/or low sleep quality (Table 1). Compared with individuals excluded from the analysis due to a lack of data on mental health status, participants were younger, had lower BMIs, a higher educational status, were more physically active, and there was a lower prevalence of current smokers and a higher proportion of occasional drinkers (Supplementary Table S1).

The background characteristics distributed across the three categories of the intake of individual polyphenol-rich and alcoholic beverages containing polyphenols are presented in Table 2 and Table 3, respectively. There was a significantly higher proportion of women among individuals consuming more tea and white wine and among those characterized by a moderate (up to 1 glass/cup a day) coffee and red wine intake, while beer was significantly more consumed by men. There was a significant, yet not clear, trend in the distribution of age groups by the level of consumption of tea, citrus fruit juice, and white wine (more consumed in middle-aged individuals), while there was a significantly higher proportion of older participants consuming coffee and red wine, and younger participants consuming more beer. Education level was slightly lower among higher coffee and white wine consumers, higher among citrus juice and beer, and with no trend among red wine consumers. Among individuals consuming more tea and citrus juice there was a significantly higher proportion of non-smokers, while among those consuming more coffee (Table 2) and red and white wine (Table 3) there were more current and former smokers compared with no consumption.

Concerning physical activity levels, there was a higher proportion of more active individuals among citrus juice, red and white wine, and beer consumers, while no clear patterns were evidenced for tea and coffee drinkers (Table 2 and Table 3). Similarly, there was a lower proportion of obese individuals among higher tea, citrus juice, red and white wine, and beer consumers, while there was a higher proportion of obesity among individuals consuming up to one cup of coffee per day. Finally, adherence to the Mediterranean diet was significantly differently distributed across categories of some polyphenol-rich beverages consumption: higher adherence was found among higher tea, citrus juice, and white wine consumers, and in those consuming up to one glass of red wine per day.

**Figure 1:**
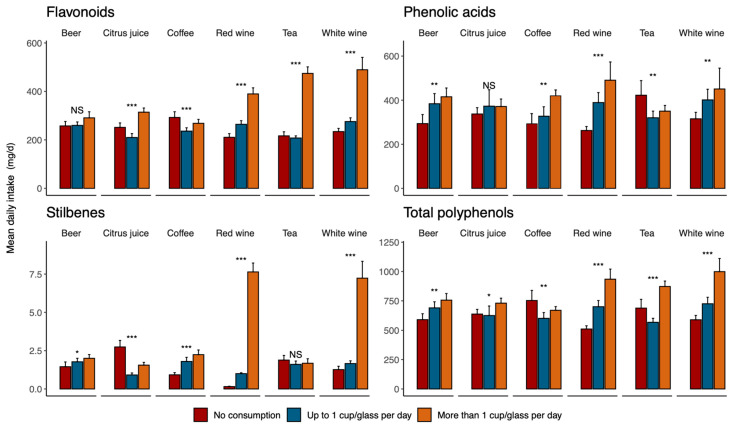
Distribution of mean intake of total and specific classes of polyphenols by intake of polyphenol-rich beverages. * *p* < 0.05; ** *p* = 0.001; *** *p* < 0.001; NS—not significant.

Figure 1 describes the distribution of the mean intake of total and specific classes of polyphenols by the intake of polyphenol-rich beverages. While the total polyphenol intake significantly increased with the intake of red and white wine and beer, there was a lower intake among moderate consumers of citrus juice and tea, and an unclear trend when considering the coffee intake categories, being made up of individuals who reported no consumption of coffee but who had the highest intakes of total polyphenols. This pattern substantially changes when considering individual polyphenol groups. For instance, higher flavonoid intake was found among higher tea, and red and white wine drinkers; higher phenolic acid intake was found among coffee, red and white wine, and beer drinkers; the mean intake of stilbenes was again higher among red and white wine drinkers, but also in those consuming more coffee, despite the overall quantity being lower than in the former.

**Figure 2:**
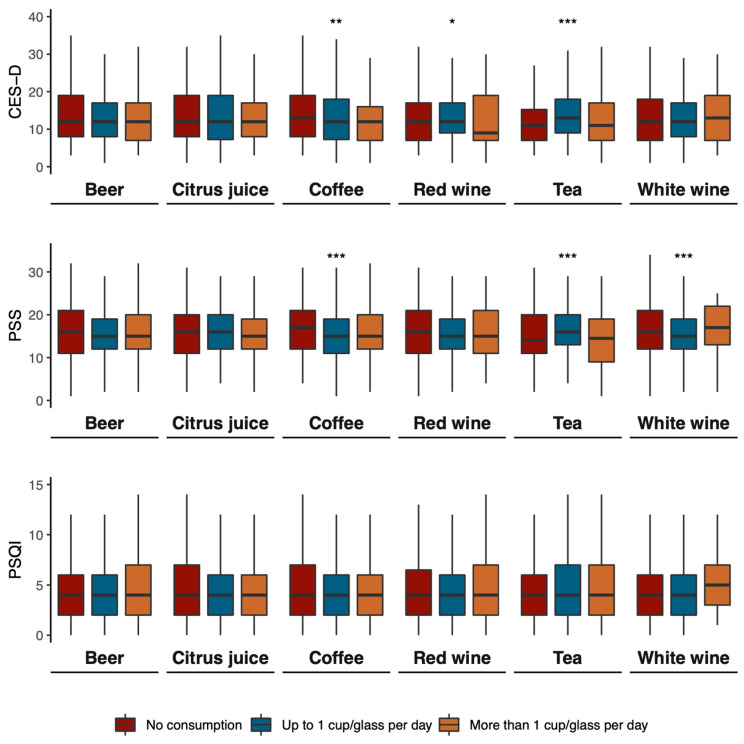
Mean scores of CES-D, PSS, and PSQI by consumption of polyphenol-rich beverages. * *p* < 0.05; ** *p* = 0.001; *** *p* < 0.001.

Figure 2 presents the distribution of the PSQI, CES-D, and PSS scores by categories of polyphenol-rich beverage consumption. Overall, there were no significant median differences between the groups.

Table 4 shows the association between the intake of individual polyphenol-rich and alcoholic beverages containing polyphenols and mental health outcomes investigated in this study. While none of the unadjusted models resulted in significant associations for the highest intake of beverages compared with the lowest intake, the multivariate model adjusted for background covariates and Mediterranean diet revealed that individuals with a moderate intake (up to 1 cup/glass per day) of coffee and tea were less likely to have high perceived stress (OR = 0.61, 95% CI: 0.45–0.84) and depressive symptoms (OR = 0.56, 95% CI: 0.39–0.80), respectively. Moreover, regular coffee and moderate/regular red wine drinkers were less likely to have depressive symptoms (OR = 0.72, 95% CI: 0.54–0.95 and OR = 0.74, 95% CI: 0.54–0.99, respectively). No significant associations were retrieved for the intake of polyphenol-rich and alcoholic beverages and sleep quality.

## 4. Discussion

The results from this study show that beverages rich in polyphenols are associated, to various extents, with the mental health status of southern Italian individuals. While no significant results were found for sleep quality, other outcomes such as perceived stress and depressive symptoms were inversely associated with different coffee, tea, and red wine intakes. Interestingly, some associations (i.e., for tea or red wine) were not dose-dependent but occurred for moderate intakes (such as, 1 cup/glass per day), suggesting that a higher intake of one beverage did not necessarily imply the highest intake of polyphenols and that a moderate intake versus no intake could have some benefits, while excess consumption could be jeopardized by other compounds (i.e., alcohol, caffeine, etc.) that may counteract the beneficial effects. To our knowledge, this is the first study investigating the relation between polyphenol-rich beverages and mental health outcomes in Italy.

The results reported in this study are generally in line with the emerging existing scientific literature published on this topic. A meta-analysis of observational studies including a total of 346,913 individuals and 8146 cases of depression showed a J-shaped relation between coffee consumption and the risk of depression, with the lowest risk registered at 400 mL/d [37]. Another meta-analysis including 22,817 participants with 4743 cases of depression showed a linear relation between tea consumption and depression risk [38]. However, no data was previously published on Italian individuals recruited from the general population: a report from the InChianti (*Invecchiare in Chianti*) study conducted on 1058 Italian participants (aged 20–102 years) reported no association between either tea or coffee consumption and symptoms of depression [39], while two other studies conducted on a sample of 300 non-demented elderly Italian subjects with subcortical ischemic vascular disease showed that daily moka pot coffee intake was associated with a higher mood status, with a significant dose-response association even for moderate consumption [40,41]. A recent study from the UK Biobank including 402,290 participants showed that moderate coffee consumption, alongside adequate sleep quality, would reduce the risk of mental disorders, such as depressive and anxiety disorders [42]. When considering the wider aspects of mood and psychological well-being, a recent study from the Nurses’ Health Study involving multiple assessments from 1992–2000 (N = 44,449) and the 2004–2012 (N = 36,729) data collections reported that moderate coffee intake was associated with a greater likelihood of sustained optimism [43]. However, in another study conducted on Japanese auto factory workers (n = 5256), coffee consumption and smoking status was not significantly related to psychological well-being [44].

Concerning wine consumption, the only Italian data was published from the InChianti (Invecchiare in Chianti) study conducted on 1362 participants aged 18–102 years (mean age 68 y) followed-up for 9 years. The study reported that a typical Italian diet characterized by vegetables, olive oil, grains, fruits, fish, and moderate meat and wine consumption was consistently inversely associated with depressive symptoms both at baseline and at follow-up [45]. A more recent study conducted on 5505 participants (55 to 80 y) of the PREDIMED Trial (Spain), followed-up for up to 7 years, showed that moderate alcohol intake (5 to 15 g/day) from wine (two to seven drinks/week) was significantly associated with a lower risk of incident depression [46]. In another study conducted on 13,619 university graduates (mean age 38 y) participating in a Spanish prospective epidemiological cohort (the SUN Project) followed-up for 10 years there was a U-shaped relationship between total alcohol intake and depression risk among women, although no apparent association with a specific type of alcoholic beverage was reported [47]. Similarly to the PREDIMED study, a moderate alcohol intake (5 to 15 g/day) was associated with a lower risk of depression [47]. However, another study conducted on 5299 community-dwelling older adults from the ELSA (English Longitudinal Study of Ageing) and Seniors-ENRICA cohorts (Study on Nutrition and Cardiovascular Risk in Spain) reported that moderate drinkers showed comparable scores on psychological tests with no different risk of being clinically diagnosed with depression [48]. Furthermore, studies on secondary prevention of depression following a primary major disease showed the potential beneficial effects of moderate alcohol (especially from wine) consumption. Two studies on colorectal cancer survivors showed that moderate alcohol drinkers (<7 drinks per week) were less likely to have depressive symptoms and reported having better health-related quality of life compared with abstainers [49,50]. Moreover, similar findings were reported in 6973 patients enrolled in the Gruppo Italiano per lo Studio della Sopravvivenza nell’Insufficienza Cardiaca-Heart Failure (GISSI-HF) trial in which those with more frequent wine consumption had less frequent symptoms of depression, a better perception of health status, and lower plasma levels of the biomarkers of vascular inflammation [51].

Polyphenol-rich beverages are a source of fluids and may contribute to the daily amount of ingested water [52]. However, the specific types of beverages (i.e., espresso/stove coffee), the scarcity of intake in the population under study (i.e., tea), and findings on moderate rather than large amounts (i.e., wine) suggest that the potential beneficial effects may rely on other qualitative aspects of such beverages rather than the mere content of water. Each beverage is, in fact, a source of different polyphenol compounds: coffee is rich in hydroxycinnamic acids, such as chlorogenic acid, ferulic acid, and caffeic acid [53], while tea is characterized by a high content of flavonoids, such as epigallocatechin-3-gallate, epigallocatechin, epicatechin-3-gallate, and epicatechin [54]. Finally, red wine is a major source of resveratrol, a stilbene richly contained in grapes [55]. All these compounds have been demonstrated to potentially exert, to a various extent, anti-inflammatory effects in the central nervous system [56]. The bioactive constituents of coffee are able to modulate the parameters of neuroinflammation through anti-inflammatory and antioxidant properties [57]. Tea polyphenols are also known to be potent anti-inflammatory agents (i.e., via down-regulation of NF-κB signaling) and effective modulators of dopaminergic activity [58]. The main biological mechanisms identified to potentially explain the effects of resveratrol (and red wine) on brain health include the regulation of the hypothalamic–pituitary–adrenal (HPA) axis, an increase in brain-derived neurotrophic factor (BDNF) and neurogenesis, and anti-inflammatory effects [59]. Emerging evidence also involves the potential effects of polyphenols on the gut–brain axis, intended as a bi-directional communication network connecting the central nervous system to the gut through a variety of pathways, including the nervous system (i.e., vagus nerve), immune system, neuroendocrine pathways, and an alteration in the gut-microbiota [60,61]. Polyphenols are largely metabolized by the gut microbiota, leading to the production of metabolites that exert an important support on the functional ecology of symbiotic partners, which in turn may affect the host physiology [62,63]. Polyphenols derived from coffee [64], tea [65], and wine [66,67] have been reported to be able to modulate the gut microbiota in favor of strains, such as Lactobacillus spp. and Bifidobacterium spp., that positively affect mental health. The mechanisms through which the modulation of gut microbiota may exert favorable effects on mental health disorders are only partially elucidated, but the integrity of the colonic mucosa, nervous impulses transmission through the vagus nerve, and dopaminergic/serotonergic systems have been found to be related to microbiota [68,69,70]. Moreover, polyphenols derived from beverages may also increase certain gut microbiota strains, such as Eubacterium, Ruminococcaceae, and Roseburia [71,72,73,74,75], which are known to improve the production of short-chain fatty acids (SCFAs), such as butyrate, acetate, and propionate [76], which have been shown to be able to cross the blood brain barrier and exhibit antidepressant and anxiolytic effects by ameliorating psychosocial stress-induced alterations in behavior [77,78].

The results of the present study should be considered in light of some limitations. Firstly, the cross-sectional design of the study does not allow for the establishment of causality but only associations. Moreover, given the cross-sectional nature of the study, the conclusions might be biased by reverse causality. Second, although various factors have been considered as potential confounders, we cannot rule out the possibility of residual confounding from unmeasured variables (i.e., social integration, family history of specific diseases, other environmental factors, etc.). Moreover, compared with non responders, the participants included in this analysis had a higher educational status and healthier lifestyle behaviors: thus, the results of this study may not necessarily apply to the totality of the population but might be limited to some subgroups. Third, although the use of FFQ is widely adopted in observational studies, the dietary exposure assessment may be affected by recall bias and over- or underestimation. Finally, exposure to dietary factors may identify features of dietary patterns or larger clusters of lifestyle behaviors and the variables of interest may not be sufficient to exert the expected effects alone.

## 5. Conclusions

In conclusion, the results of the present study suggest that polyphenol-rich beverages may be associated with mental health, specifically, the consumption of coffee, tea, and red wine was associated with lower depressive symptoms and perceived stress. Considering that the overall consumption of such beverages in this sample was moderate, the findings reported in this study suggest that the retrieved associations with better mental health outcomes are more likely to be considered for the moderate consumption of a variety of polyphenol-rich beverages rather than higher intake of a single one. Additional studies are needed to better describe the optimal patterns of consumption of polyphenol-rich beverages and whether these may differ across populations with different dietary patterns. Moreover, further research exploring how the polyphenol-rich beverages impact brain health from a mechanistic point of view is highly warranted.

## Figures and Tables

**Table 1:** Background characteristics of the study sample (n = 1572).

	n (%)
**Age (years), mean (SD)**	46.6 (17.2)
**Sex**	
Men	660 (42.0)
Women	912 (58.0)
**BMI, mean (SD)**	25.6 (4.4)
**Smoking status**	
Current	384 (24.4)
Former	178 (11.3)
Never	1010 (64.3)
**Educational level**	
Low	457 (29.0)
Medium	644 (41.0)
High	471 (30.0)
**Physical activity level**	
Low	277 (17.6)
Medium	779 (49.5)
High	517 (32.9)
**Alcohol consumption**	
None	289 (18.4)
Occasional (0.1–12 g/d)	1005 (63.9)
Regular (>12 g/d)	278 (17.7)
**Mental health**	
Depressive symptoms	509 (32.4)
Perceived stress	486 (30.9)
Low sleep quality	516 (32.8)
**Total energy intake (kcal/d), mean (SD)**	2086.4 (849.7)

**Table 2:** Background characteristics of the study sample by consumption of individual non-alcoholic polyphenol-rich beverages.

	Tea			Coffee			Citrus Juice		
	No Intake (n = 544)	Up to 1 Cup/d (n = 708)	>1 Cup/d (n = 320)	No Intake (n = 453)	Up to 1 Cup/d (n = 502)	More Than 1 Cup/d (n = 617)	No Intake (n = 465)	Up to 1 Glass/d (n = 462)	>1 Glass/d (n = 645)
**Sex, n (%)**									
Male	252 (46.3)	305 (43.1)	103 (32.2)	205 (45.3)	180 (35.9)	275 (44.6)	177 (38.1)	197 (42.6)	286 (44.3)
Female	292 (53.7)	403 (56.9)	217 (67.8) **	248 (54.7)	322 (64.1)	342 (55.4) *	288 (61.9)	265 (57.4)	359 (55.7)
**Age group, n (%)**									
<30	103 (18.9)	140 (19.8)	73 (22.8)	116 (25.6)	86 (17.1)	114 (18.5)	94 (20.2)	94 (20.3)	128 (19.8)
30–39	118 (21.7)	123 (17.4)	56 (17.5)	101 (22.3)	85 (16.9)	111 (18.0)	73 (15.7)	92 (19.9)	132 (20.5)
40–49	106 (19.5)	129 (18.2)	76 (23.8)	81 (17.9)	103 (20.5)	127 (20.6)	89 (19.1)	92 (19.9)	130 (20.2)
50–59	72 (13.2)	114 (16.1)	57 (17.8)	63 (13.9)	70 (13.9)	110 (17.8)	60 (12.9)	96 (20.8)	87 (13.5)
60–69	83 (15.3)	111 (15.7)	37 (11.6)	50 (11.0)	87 (17.3)	94 (15.2)	80 (17.2)	39 (8.4)	112 (17.4)
>69	62 (11.4)	91 (12.9)	21 (6.6) *	42 (9.3)	71 (14.1)	61 (9.9) **	69 (14.8)	49 (10.6)	56 (8.7) **
**Educational level, n (%)**									
Low	165 (30.3)	207 (29.2)	85 (26.6)	110 (24.3)	168 (33.5)	179 (29.0)	175 (37.6)	128 (27.7)	154 (23.9)
Medium	237 (43.6)	274 (38.7)	133 (41.6)	203 (44.8)	180 (35.9)	261 (42.3)	169 (36.3)	186 (40.3)	289 (44.8)
High	142 (26.1)	227 (32.1)	102 (31.9)	140 (30.9)	154 (30.7)	177 (28.7) *	121 (26.0)	148 (32.0)	202 (31.3) **
**Smoking status, n (%)**									
Non-smoker	335 (61.6)	446 (63.0)	229 (71.6)	341 (75.3)	314 (62.5)	355 (57.5)	274 (58.9)	306 (66.2)	430 (66.7)
Current smoker	129 (23.7)	179 (25.3)	76 (23.8)	74 (16.3)	132 (26.3)	178 (28.8)	111 (23.9)	116 (25.1)	157 (24.3)
Former smoker	80 (14.7)	83 (11.7)	15 (4.7) **	38 (8.4)	56 (11.2)	84 (13.6) **	80 (17.2)	40 (8.7)	58 (9.0) **
**Physical activity level, n (%)**									
Low	93 (17.1)	136 (19.3)	48 (15.1)	89 (19.7)	97 (19.4)	91 (14.7)	104 (22.4)	89 (19.4)	84 (13.0)
Medium	262 (48.2)	331 (46.9)	181 (56.9)	200 (44.3)	255 (51.0)	319 (51.7)	260 (55.9)	223 (48.7)	291 (45.1)
High	189 (34.7)	239 (33.9)	89 (28.0) *	162 (35.9)	148 (29.6)	207 (33.5) *	101 (21.7)	146 (31.9)	270 (41.9) **
**BMI categories, n (%)**									
Normal	223 (44.2)	298 (45.2)	179 (61.9)	217 (53.3)	231 (48.7)	252 (44.1)	185 (44.8)	217 (49.9)	298 (49.3)
Overweight	181 (35.8)	251 (38.1)	82 (28.4)	139 (34.3)	153 (32.3)	222 (38.8)	138 (33.4)	142 (32.6)	234 (38.7)
Obese	101 (20.0)	110 (16.7)	28 (9.7) **	51 (12.5)	90 (19.0)	98 (17.1) *	90 (21.8)	76 (17.5)	73 (12.1) **
**Mediterranean diet adherence, n (%)**									
Low	310 (57.0)	401 (56.6)	147 (45.9)	247 (54.5)	279 (55.6)	332 (53.8)	276 (59.4)	288 (62.3)	294 (45.6)
Medium	186 (34.2)	240 (33.9)	131 (40.9)	153 (33.8)	180 (35.9)	224 (36.3)	174 (37.4)	138 (29.9)	245 (38.0)
High	48 (8.8)	67 (9.5)	42 (13.1) *	53 (11.7)	43 (8.6)	61 (9.9)	15 (3.2)	36 (7.8)	106 (16.4) **

* indicates *p* < 0.05 for Chi-square analysis. ** indicates *p* < 0.001 for Chi-square analysis.

**Table 3:** Background characteristics of the study sample by consumption of individual alcoholic polyphenol-rich beverages.

	Red Wine			White Wine			Beer		
	No Intake (n = 531)	Up to 1 Glass/d (n = 803)	>1 Glass/d (n = 238)	No Intake (n = 764)	Up to 1 Glass/d (n = 737)	>1 Glass/d (n = 71)	No in Taken (n = 492)	Up to 1 Glass/d (n = 775)	>1 Glass/d (n = 305)
**Sex, n (%)**									
Male	200 (37.7)	357 (44.5)	103 (43.3)	301 (39.4)	339 (46.0)	20 (28.2)	171 (34.8)	332 (42.8)	157 (51.5)
Female	331 (62.3)	446 (55.5)	135 (56.7) *	463 (60.6)	398 (54.0)	51 (71.8) *	321 (65.2)	443 (57.2)	148 (48.5) **
**Age group, n (%)**									
<30	119 (22.4)	176 (21.9)	21 (8.8)	168 (22.0)	145 (19.7)	3 (4.2)	85 (17.3)	160 (20.6)	71 (23.3)
30–39	114 (21.5)	145 (18.1)	38 (16.0)	137 (17.9)	142 (19.3)	18 (25.4)	80 (16.3)	157 (20.3)	60 (19.7)
40–49	91 (17.1)	173 (21.5)	47 (19.7)	147 (19.2)	148 (20.1)	16 (22.5)	84 (17.1)	165 (21.3)	62 (20.3)
50–59	61 (11.5)	151 (18.8)	31 (13.0)	95 (12.4)	138 (18.7)	10 (14.1)	70 (14.2)	110 (14.2)	63 (20.7)
60–69	78 (14.7)	94 (11.7)	59 (24.8)	127 (16.6)	90 (12.2)	14 (19.7)	89 (18.1)	108 (13.9)	34 (11.1)
>69	68 (12.8)	64 (8.0)	42 (17.6) **	90 (11.8)	74 (10.0)	10 (14.1) *	84 (17.1)	75 (9.7)	15 (4.9) **
**Educational level, n (%)**									
Low	186 (35.0)	176 (21.9)	95 (39.9)	246 (32.2)	183 (24.8)	28 (39.4)	170 (34.6)	212 (27.4)	75 (24.6)
Medium	210 (39.5)	358 (44.6)	76 (31.9)	293 (38.4)	327 (44.4)	24 (33.8)	191 (38.8)	312 (40.3)	141 (46.2)
High	135 (25.4)	269 (33.5)	67 (28.2) **	225 (29.5)	227 (30.8)	19 (26.8) *	131 (26.6)	251 (32.4)	89 (29.2) *
**Smoking status, n (%)**									
Non-smoker	367 (69.1)	509 (63.4)	134 (56.3)	515 (67.4)	458 (62.1)	37 (52.1)	317 (64.4)	498 (64.3)	195 (63.9)
Current smoker	107 (20.2)	219 (27.3)	58 (24.4)	163 (21.3)	201 (27.3)	20 (28.2)	106 (21.5)	203 (26.2)	75 (24.6)
Former smoker	57 (10.7)	75 (9.3)	46 (19.3) **	86 (11.3)	78 (10.6)	14 (19.7) *	69 (14.0)	74 (9.5)	35 (11.5)
**Physical activity level, n (%)**									
Low	115 (21.7)	127 (15.9)	35 (14.7)	155 (20.3)	118 (16.1)	4 (5.6)	111 (22.6)	121 (15.7)	45 (14.8)
Medium	275 (52.0)	377 (47.1)	122 (51.3)	402 (52.8)	328 (44.6)	44 (62.0)	260 (53.0)	365 (47.3)	149 (48.9)
High	139 (26.3)	297 (37.1)	81 (34.0) **	205 (26.9)	289 (39.3)	23 (32.4) **	120 (24.4)	286 (37.0)	111 (36.4) **
**BMI categories, n (%)**									
Normal	251 (51.6)	359 (48.3)	90 (40.2)	343 (48.2)	325 (48.4)	32 (46.4)	228 (49.9)	343 (47.7)	129 (46.6)
Overweight	142 (29.2)	279 (37.6)	93 (41.5)	234 (32.9)	247 (36.8)	33 (47.8)	140 (30.6)	256 (35.6)	118 (42.6)
Obese	93 (19.1)	105 (14.1)	41 (18.3) **	135 (19.0)	100 (14.9)	4 (5.8) *	89 (19.5)	120 (16.7)	30 (10.8) *
**Mediterranean diet adherence, n (%)**									
Low	344 (64.8)	420 (52.3)	94 (39.5)	466 (61.0)	363 (49.3)	29 (40.8)	281 (57.1)	416 (53.7)	161 (52.8)
Medium	155 (29.2)	282 (35.1)	120 (50.4)	243 (31.8)	284 (38.5)	30 (42.3)	176 (35.8)	273 (35.2)	108 (35.4)
High	32 (6.0)	101 (12.6)	24 (10.1) **	55 (7.2)	90 (12.2)	12 (16.9) **	35 (7.1)	86 (11.1)	36 (11.8)

* indicates *p* < 0.05 for Chi-square analysis. ** indicates *p* < 0.001 for Chi-square analysis.

**Table 4:** Association between polyphenol-rich beverages consumption and mental health outcomes in the study sample.

	Sleep Quality, OR (95% CI)			Perceived Stress, OR (95% CI)			Depressive Symptoms, OR (95% CI)		
	No Consumption	Up to 1 Cup-Glass/d	More Than 1 Cup-Glass/d	No Consumption	Up to 1 Cup-Glass/d	More than 1 Cup-Glass/d	No Consumption	Up to 1 Cup-Glass/d	More than 1 Cup-Glass/d
**Tea**									
Model 1 ^a^	1	0.79 (0.57–1.07)	0.95 (0.71–1.26)	1	0.97 (0.72–1.30)	2.23 (0.90–5.50)	1	0.60 (0.43–0.84) *	1.25 (0.94–1.66)
Model 2 ^b^	1	0.77 (0.55–1.08)	0.89 (0.65–1.20)	1	1.03 (0.74–1.42)	2.41 (0.97–5.94)	1	0.58 (0.40–0.82) *	1.09 (0.80–1.49)
Model 3 ^c^	1	0.72 (0.51–1.00)	0.84 (0.62–1.14)	1	1.03 (0.74–1.43)	2.42 (0.98–5.99)	1	0.56 (0.39–0.80) *	1.05 (0.77–1.43)
**Coffee**									
Model 1 ^a^	1	0.95 (0.71–1.26)	0.88 (0.67–1.17)	1	0.69 (0.51–0.92) *	0.86 (0.65–1.14)	1	1.00 (0.75–1.34)	0.77 (0.58–1.03)
Model 2 ^b^	1	0.93 (0.68–1.27)	0.85 (0.63–1.15)	1	0.61 (0.45–0.84) *	0.88 (0.65–1.20)	1	0.94 (0.69–1.29)	0.76 (0.56–1.05)
Model 3 ^c^	1	0.96 (0.70–1.30)	0.90 (0.66–1.22)	1	0.61 (0.45–0.84) *	0.89 (0.65–1.21)	1	0.91 (0.62–1.33)	0.72 (0.54–0.95) *
**Citrus juice**									
Model 1 ^a^	1	0.98 (0.72–1.33)	1.01 (0,76–1.34)	1	1.01 (0.74–1.36)	0.79 (0.60–1.05)	1	1.04 (0.76–1.41)	0.76 (0.56–1.01)
Model 2 ^b^	1	1.13 (0.81–1.56)	1.12 (0.82–1.52)	1	0.94 (0.68–1.30)	0.85 (0.62–1.15)	1	1.01 (0.73–1.41)	0.78 (0.57–1.07)
Model 3 ^c^	1	1.06 (0.76–1.48)	1.13 (0.83–1.54)	1	0.92 (0.66–1.28)	0.85 (0.62–1.16)	1	1.01 (0.73–1.41)	0.79 (0.57–1.08)
**Red wine**									
Model 1 ^a^	1	1.06 (0.72–1.57)	0.98 (0.68–1.42)	1	0.96 (0.65–1.42)	1.00 (0.69–1.43)	1	0.75 (0.51–1.12)	0.88 (0.60–1.27)
Model 2 ^b^	1	0.93 (0.61–1.40)	0.92 (0.62–1.37)	1	0.81 (0.53–1.23)	0.84 (0.56–1.25)	1	0.70 (0.46–1.07)	0.72 (0.48–1.08)
Model 3 ^c^	1	0.86 (0.59–1.26)	0.80 (0.56–1.13)	1	0.86 (0.58–1.26)	0.75 (0.53–1.07)	1	0.60 (0.40–0.88) *	0.74 (0.54–0.99) *
**White wine**									
Model 1 ^a^	1	0.94 (0.70–1.26)	1.40 (0.78–2.48)	1	0.68 (0.51–0.92) *	1.29 (0.69–2.43)	1	1.00 (0.74–1.35)	1.09 (0.60–1.99)
Model 2 ^b^	1	0.90 (0.66–1.23)	1.34 (0.74–2.43)	1	0.76 (0.56–1.04)	1.06 (0.55–2.04)	1	1.32 (0.95–1.83)	1.15 (0.87–1.53)
Model 3 ^c^	1	1.25 (0.97–1.61)	0.71 (0.47–1.07)	1	0.96 (0.74–1.25)	0.82 (0.56–1.21)	1	1.17 (0.89–1.52)	1.02 (0.79–1.20)
**Beer**									
Model 1 ^a^	1	1.04 (0.79–1.36)	1.43 (1.01–2.01)	1	1.15 (0.85–1.45)	1.19 (0.84–1.69)	1	0.87 (0.66–1.15)	0,78 (0.54–1.12)
Model 2 ^b^	1	0.95 (0.71–1.28)	1.31 (0.90–1.90)	1	1.18 (0.88–1.58)	1.38 (0.94–2.02)	1	0.91 (0.68–1.23)	0.93 (0.63–1.37)
Model 3 ^c^	1	0.92 (0.69–1.23)	1.26 (0.87–1.83)	1	1.15 (0.87–1.54)	1.34 (0.92–1.95)	1	0.93 (0.69–1.24)	0.95 (0.65–1.40)

^a^ Adjusted for total energy intake and all beverages investigated. ^b^ Adjusted as model 1 plus age, sex, educational status, smoking status, physical activity level. ^c^ Adjusted as model 2 plus adherence to the Mediterranean diet. * indicates *p* < 0.05 for logistic regression analysis.

## Data Availability

The data that support the findings of this study are available upon reasonable request.

## Supplementary Materials

The following supporting information can be downloaded at: https://www.mdpi.com/article/10.3390/antiox12020272/s1, Table S1: Differences in background characteristics between included and excluded sample.
