# Supplementary material for: Polyphenol-Rich Beverages and Mental Health Outcomes
Source: Antioxidants (Basel). 2023 Jan 25;12(2):272. doi: 10.3390/antiox12020272 (PMC9952274; doi:10.3390/antiox12020272)
Supplement: Supplementary file 1 [file antioxidants-12-00272-s001.zip › antioxidants-2163249-supplementary.pdf]

Table S1. Differences in background characteristics between included and excluded sample.

	Included	Excluded	P-value
**Age (years), mean (SD)**	46.6 (17.2)	55.4 (17.2)	<0.001
**Sex**			0.398
Men	660 (42.0)	144 (39.6)	
Women	912 (58.0)	220 (60.4)	
**BMI, mean (SD)**	25.6 (4.4)	26.6 (4.8)	0.001
**Smoking status**			<0.001
Current	384 (24.4)	81 (22.3)	
Former	178 (11.3)	98 (26.9)	
Never	1010 (64.2)	185 (50.8)	
**Educational level**			<0.001
Low	457 (29.0)	240 (65.9)	
Medium	644 (41.0)	76 (20.9)	
High	471 (30.0)	48 (13.2)	
**Physical activity level**			<0.001
Low	277 (17.6)	52 (32.5)	
Medium	779 (49.5)	82 (51.3)	
High	517 (32.9)	26 (16.3)	
**Alcohol consumption**			0.007
None	289 (18.4)	86 (23.6)	
Occasional (0.1-12 g/d)	1005 (63.9)	201 (55.1)	
Regular (>12 g/d)	278 (17.7)	78 (21.4)	
**Total energy intake (kcal/d), mean (SD)**	2086.4 (849.7)	2230.6 (652.4)	0.002
